# Implementation of an intervention to improve the adoption of asthma self-management practices in Peru: Asthma Implementation Research (AIRE) randomized trial study protocol

**DOI:** 10.1186/s13063-020-4207-5

**Published:** 2020-05-04

**Authors:** Elisa D. Romani, Trishul Siddharthan, Nair Lovatón, Carol C. Alvítez-Luna, Oscar Flores-Flores, Suzanne L. Pollard

**Affiliations:** 1 Biomedical Research Unit, Asociación Benéfica PRISMA, Carlos González 251, San Miguel, Lima Peru; 2 Center for Non-Communicable Disease Research and Training, Johns Hopkins University, Baltimore, MD USA; 3 Division of Pulmonary and Critical Care, School of Medicine, Johns Hopkins University, 1830 E. Monument Street, Ste 516, Baltimore, MD USA; 4 Pediatric Pulmonology Unit, Cayetano Heredia Hospital, Av. Honorio Delgado 262, San Martín de Porres, Baltimore, Peru; 5 Universidad de San Martin de Porres, Facultad de Medicina Humana, Centro de Investigación del Envejecimiento (CIEN), Lima, Peru; 6 Universidad Cientifica del Sur, Facultad de Ciencias de la Salud, Lima, Peru; 7 Department of International Health, Bloomberg School of Public Health, Johns Hopkins University, Baltimore, MD USA

**Keywords:** Asthma, Asthma self-management practices, Asthma action plan, Asthma community home visit, Implementation Science, Randomized controlled trial

## Abstract

**Introduction:**

Asthma is the most common chronic disease among children worldwide, with 80% of asthma-related deaths occurring in low- and middle-income countries (LMICs). While evidence-based guidelines exist for asthma treatment and management, adoption of guideline-based practices is low in high-income country and LMIC settings alike. While asthma prevalence among children and adolescents in Lima, Peru is in the range of 13%–19.6%, our data suggest that < 5% of children in low-resource communities are currently taking guideline-based therapies. There is an urgent need for effective, locally tailored solutions to address the asthma treatment gap in low-income communities in Peru.

**Methods:**

This study aims to develop and test a locally adapted intervention package to improve adoption of self-management practices and utilization of preventive health services for asthma among children in Lima Norte. The intervention package was designed using a systematic, theory-based framework (Capability, Opportunity, Motivation – Behavior Framework) and is rooted in a multi-phased formative research approach. The main study design is an individually randomized implementation-effectiveness hybrid trial enrolling 110 children aged 5–17 years with asthma and their caregivers. Families allocated to the treatment group receive the supported self-management intervention package, while families allocated to the control group receive the standard of care plus asthma education. We will follow participants monthly for six months and evaluate asthma control (Asthma Control Test), healthcare utilization, and medication adherence (Adherence to Refills and Medications Scale). Disease-specific quality of life for children (Pediatric Asthma Quality of Life Questionnaire) and caregivers (Pediatric Asthma Caregiver’s Quality of Life Questionnaire) will be evaluated at baseline, 3 months, and 6 months. We will also evaluate acceptability, feasibility, and fidelity of the intervention using mixed methods approaches.

**Discussion:**

The long-term goal of this study is to disseminate locally appropriate asthma management strategies in LMIC settings. This study will contribute to the body of knowledge surrounding approaches for developing and evaluating intervention strategies for asthma using systematic, theory-based approaches grounded in local context. Such strategies have the potential to inform the development and adaptation of appropriate and scalable solutions for asthma management in LMIC settings.

**Trial registration:**

ClinicalTrials.gov, NCT03986177. Registered on 14 June 2019.

## Background

Asthma is the most common chronic disease among children, with an estimated 334 million individuals affected worldwide [1]. Asthma has far-reaching implications for both the individual and society, leading to impaired child development and education, impeded job training and employment, and increasing healthcare expenditures at the national level. While asthma is a well-recognized public health issue in high-income countries, there remains insufficient focus on addressing the asthma epidemic in low- and middle-income countries (LMICs), where > 80% of asthma-related deaths occur [2].

Peru has one of the highest asthma burdens in the world, with prevalence estimates as high as 19.6% in the capital city of Lima [3]. Robinson et al. demonstrated that the prevalence of current wheeze among children aged 13–15 years was 12%, and the prevalence of physician-diagnosed asthma was 13% [4] among two peri-urban communities in Lima. Importantly, asthma was poorly controlled in these communities: 17.3% of the cohort of children had severe persistent asthma; 17.5% had moderate persistent asthma; 43.7% had mild persistent asthma; and 21.5% had intermittent asthma, as per NAEPP-3/ERS guidelines [5].

National and international guidelines exist for the diagnosis, treatment, and self-management of asthma. The core components of guideline-based self-management for asthma include comprehensive asthma education, adherence to evidence-based asthma medications, environmental trigger abatement, and regular follow-up with a health provider [6, 7]. However, our preliminary research demonstrates that in peri-urban communities of Peru, < 5% of children with persistent asthma as per NAEPP-3 guidelines are currently taking guideline-based medications for asthma [5, 6], for a treatment gap of 95% (unpublished data). Furthermore, only 12.8% of children with asthma reported having a doctor that continually monitors their disease.

Supported self-management has been shown to be effective and feasibly delivered in several settings and populations [8]. However, there is a paucity of research regarding the development and testing of interventions to improve asthma self-management in LMIC settings, which experience unique or exacerbated barriers to receiving evidence-based care. Reviews show that strategies that are culturally and linguistically appropriate, take into account different learning styles, provide social support, and address key contextual barriers are the most effective [8]. Furthermore, use of theory-based, systematic approaches grounded in local knowledge and context are critical to the development of effective intervention strategies [9].

The main objective of the Asthma Implementation Research (AIRE) study is to develop, test, and evaluate a locally adapted intervention package designed to improve adoption of asthma self-management practices among children living in low-resource communities in Lima, Peru. Another important goal of this study is to understand the context-specific factors that influence successful implementation of such strategies in LMIC settings such as Peru. In this protocol paper, we will describe the rationale, study design, and methods for this research, including the systematic development of the theory-based intervention package, iterative pilot testing, and an individually randomized effectiveness-implementation hybrid trial in which we follow children with asthma monthly for 6 months.

## Design and methods

### Setting

#### Health system

Peru has a decentralized healthcare system administered by five entities, the largest being the Ministry of Health (MINSA), which provides health services for 60% of the population across Peru [10]. The Seguro Integral de Salud (SIS), as part of the MINSA health system, serves individuals without any other type of insurance, including those living in poverty or other vulnerable populations (e.g. pregnant women, children, etc.).

The MINSA health system is organized into three levels. Level I, which is sub-categorized into four levels, comprises health posts (I-1, I-2) and larger health centers (I-3, I-4). Individuals who cannot be managed by the staff at Level I health centers due to disease complexity are referred through the referral system to higher levels of care. Resources and personnel available for the acute and chronic management of asthma varies by level. Lower level health posts (I-1) generally do not have personnel able to manage either acute or chronic asthma cases. Higher level health posts (I-2, I-3) are able to resolve uncomplicated acute asthma exacerbations, while level I-4 health centers are equipped with nebulizers and a 24-h emergency room to resolve asthma attacks. Level I-4 centers also often have specialists (pediatricians, pulmonologists) on staff who may provide primary care for asthma, as well as a pharmacy. Levels II and III are referral facilities that, in addition to nebulizers and 24-h emergency care, also offer specialized clinics in pediatric pulmonology, and may offer diagnostic services such as spirometry.

#### Study setting

This study will be carried out in the geographical area known as “Lima Norte” (North Lima) and will be based primarily at Cayetano Heredia Hospital, a level III referral hospital located in the district of San Martín de Porres. Cayetano Heredia Hospital is considered one of the main hospitals in northern Lima, in addition to Sergio Bernales and Carlos La Hoz Hospital. Cayetano Heredia is the hospital with the highest demand of referrals, with a catchment area comprising the districts of San Martin de Porres, Comas, Independencia, Los Olivos, and Puente Piedra. These districts are geographically adjacent; Cayetano Heredia Hospital is approximately 40 km from the center of the farthest district, Ancón, from which on public transport it may take as long as 2 h to arrive.

### Study population

For this study, we will enroll children and adolescents with asthma living in any of the following districts of Lima Norte: Ancón; Puente Piedra; Santa Rosa; San Martín de Porres; Comas; Los Olivos; Independencia; Carabayllo; and Rímac. The inclusion criteria for this study are: (1) currently living in Lima, Peru; (2) aged 5–17 years; (3) has a physician’s diagnosis of asthma; and (4) has attended the emergency room or consultation for asthma at least once in the previous 12 months. Exclusion criteria include: (1) family plans to move out of the study community within the next 6 months; (2) co-occurring chronic respiratory or cardiovascular disorders other than asthma; and (3) active tuberculosis or are currently taking tuberculosis medications.

### Study design

We will enroll 110 children in an individually randomized effectiveness-implementation hybrid trial. The objectives are: (1) to test the effectiveness of the intervention package on asthma control, adherence to preventive therapies, and quality of life; (2) to evaluate the acceptability and feasibility of implementing the intervention in this context; and (3) to identify the context-specific factors influencing successful implementation. Participants in the intervention group will receive case management from a designated nurse manager, who will provide ongoing educational, social, and self-management support during follow-up home visits (see “Intervention design”), as well as delivery and free access to prescribed preventive inhaler medications. The control group will receive comprehensive asthma education with free access to prescribed preventive inhaler medications available at a local health facility. All participants will be followed up monthly for a period of 6 months.

### Ethics

The trial protocol was approved by the Institutional Review Boards (IRB) at the Johns Hopkins School of Medicine, Asociación Benéfica PRISMA, and Cayetano Heredia Hospital. This trial is also registered with ClinicalTrials.gov (Identifier: NCT03986177).

### Formative research and intervention design

#### Formative research

**Fig. 1:**
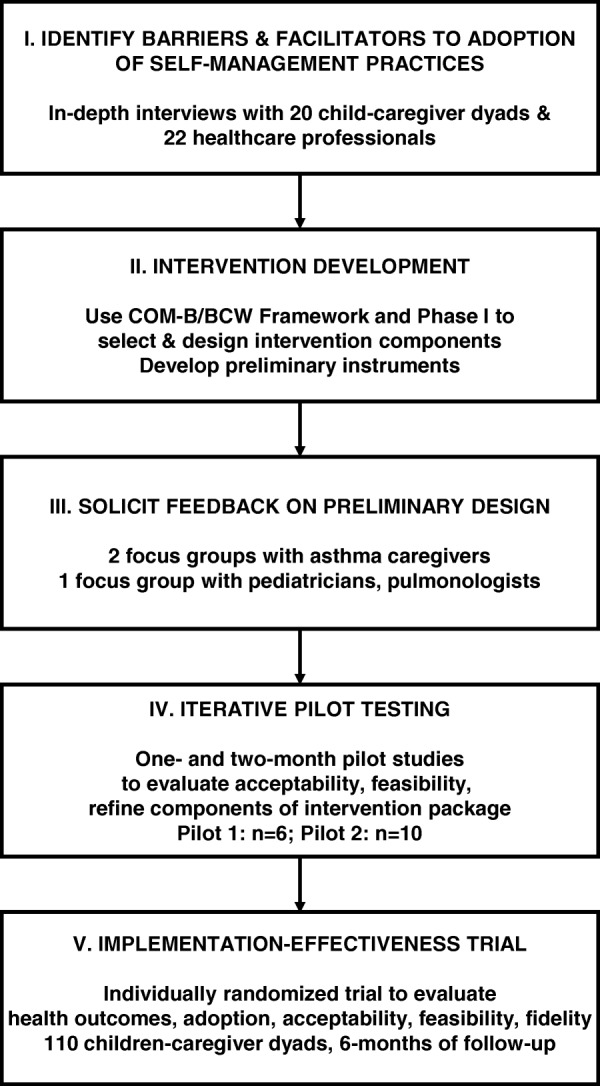
Flow diagram of Asthma Implementation Research phases

The content of the intervention was informed by a multi-phased formative research effort carried out during 2015–2019 (Fig. 1). In the first phase of formative research, we carried out in-depth interviews with key stakeholders (22 children–caregiver dyads and 20 local healthcare professionals and workers) to identify key barriers and facilitators to adoption of self-management practices in peri-urban communities of Lima.

**Fig. 2:**
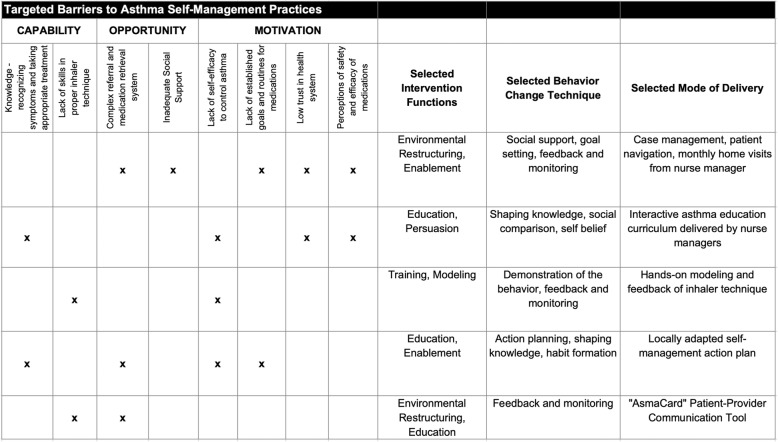
Theoretical framework and targeted barriers of the multi-component intervention package to promote adoption of asthma self-management practices

Using the data collected in this first phase, we used the Capability, Opportunity, Motivation – Behavior/Behavior Change Wheel (COM-B/BCW), a theory-based framework for designing intervention strategies derived from a synthesis of 19 existing behavior change frameworks [11], to systematically develop a preliminary design for the intervention strategy. Using this framework, we first identified, prioritized, and categorized key barriers to adoption of self-management practices for asthma in our context. Barriers were prioritized based on potential impact, perceived importance, and feasibility of addressing during this stage of research. We then used the BCW to design intervention components grounded in theory-based behavior change techniques that would target these key barriers to adoption (Fig. 2).

In the second phase of formative research, we carried out two focus groups, one with caregivers of children with asthma (*n* = 7) and another with local pulmonary physicians (*n* = 3), to solicit feedback regarding the acceptability and perceived feasibility of each intervention component. Participants also had the opportunity to review preliminary versions of the instruments and materials and provided specific feedback as to how to improve upon the design and content. We then incorporated this feedback into the next version of the design.

In the final formative phase, we conducted two iterative pilots with child–caregiver dyads (*n* = 6, *n* = 10). For these pilots, we implemented the full intervention package over a period of 1 month and 2 months, respectively. The purpose of these pilot studies was to evaluate whether the intervention was acceptable, feasible, and effective in modifying key barriers to adoption of self-management practices. Following each pilot, we conducted in-depth interviews and surveys with participating families and gathered feedback regarding the acceptability and feasibility of each component. The data from each pilot study allowed us to further refine the specifics of intervention delivery and content in this context.

#### Intervention design

For the full intervention package, child–caregiver dyads are assigned a designated nurse manager who provides monthly home visits and are available via cell phone during established hours for the period of follow-up. All instruments used in this study were created or adapted to the Peruvian context through advice and iterative feedback from local general practitioners, pulmonologists, children with asthma, and their caregivers during the formative pilot phase. For details on the content of the components and methods for adaptation to the local context, see Table 1.

**Table 1:** Intervention components, their descriptions, and local adaptations

Component	Description	Adaptations
Case management and follow-up delivered by trained nurse educators	• Personalized case management approach addressing specific needs of family/child • Monthly home visits • Patient navigation for preventive health services and follow-up • Regular check-ins by cell phone or text message with feedback on medication use	Methods: • Piloting of case management approach with caregivers and children • Expert review by local physicians Adaptations: • Modeled in part on the PROFAN program
Interactive asthma education sessions	Based on NHLBI “Breath of Life” curriculum including flipcharts, question and answers, videos, and other interactive learning activities • Asthma pathophysiology • Symptom recognition • Common treatments and medications • Trigger abatement • Motivation	Methods: • Piloting of education sessions with caregivers and children • Focus groups with caregivers • Expert review by local physicians Adaptations: • Reinforcement of key concepts over six home visits • Addition of complementary video components
“Súper Niño” comic book-style educational booklet	• Reinforces basic asthma education concepts in shorter, simpler format for children • Story format – walks children through the four tools (definition, cause, treatment, action plan) they need to manage their asthma and become a “super niño” • Reinforcement of self-efficacy and positive messaging – “Armed with these tools, I can lead a healthy, active life”	Methods: • One-month pilot with 10 children and caregivers Adaptations: • Developed by nurse educator exclusively for the Peruvian context. • Coloring book encourages younger children to engage with booklet outside of home visits
Hands-on instruction of inhaler use	• Delivered by nurse managers in the home context • Demonstration by nurse manager followed by hands-on practice by child and caregiver • Feedback and problem solving by nurse manager • Technique reinforcement provided at subsequent visits	Methods: • Consultation with local pediatric pulmonologists. • Follows Spanish aerosol therapy guidelines [12] Adaptations: • In line with local clinical practice standards and available equipment (spacers, etc.)
Locally adapted asthma action plan	• Personalized medication regimen in accordance with physician instructions • Illustrated guidance on symptom recognition and identifying “green, yellow, red” zones • Simple instructions for taking appropriate action based on zones • Includes medication diary to keep track of use of daily inhaled (preventive) medications	Methods: • Interviews and focus groups with physicians, nurses, caregivers, children Adaptations: • Simple and clear language • Bright and dynamic illustrations • Concordance with local clinical standards for care seeking
“AsmaCard”	• Unified record of clinic visits, ED visits, hospitalizations for asthma, personal triggers • ACT Questionnaire • Graph of ACT scores over time • Illustrated instructions for proper inhaler technique	Methods: • Interviews and focus groups with physicians, nurses, caregivers, children Adaptations: • Simple and clear language • Bright and dynamic illustrations • Concordance with local clinical standards for care seeking

*ACT* Asthma Control Test; *ED* Emergency Department

#### Training

Nurse managers are licensed nursing professionals with experience in community health and health education. All nurses will have had experience treating children with asthma in a clinical setting. Nurses have prior experience working in the communities in which this study will be carried out. As such, nurse managers will also provide invaluable knowledge and expertise relevant to the appropriate and effective delivery of the intervention components in this setting.

Training will include three full-day sessions. Nurse managers will be trained in several basic competencies related to asthma pathophysiology, common treatments for asthma and their mechanism of action, international and Peruvian guidelines for the diagnosis, management, and treatment of asthma, and familiarization with project goals. They will also be trained in rapport building and interpersonal communication skills, research ethics and maintaining confidentiality, maintaining personal safety, and monitoring and feedback of key self-management behaviors. Finally, nurse managers will receive hands-on training and practice in the use of all components of the intervention, including delivering asthma education, the asthma action plan, and demonstrations of inhaler technique. Refresher trainings will occur periodically throughout the study period to provide continual reinforcement and feedback.

#### Recruitment, enrollment, and retention

Children will be recruited from the pediatric pulmonary clinic at Cayetano Heredia Hospital, a level III referral hospital in Lima Norte. Children aged 5–17 years identified by physicians as meeting eligibility criteria will be referred to on-site study personnel and screened to confirm eligibility using a questionnaire. Before enrollment, study personnel will explain the goals of the study, what the study entails on the part of participating children and caregivers, and then asked if they are interested in participating. After answering any questions or concerns, we will obtain written informed consent from all caregivers and written informed assent from children before enrollment. On the consent form, participants will be asked if they agree to use of their data should they choose to withdraw from the trial. Participants will also be asked for permission for the research team to share relevant data with people from the universities taking part in the research or from regulatory authorities, where relevant. This trial does not involve collecting biological specimens for storage. To achieve maximum retention, we will offer inhaler medications to all study participants regardless of treatment assignment for the duration of the follow-up. We will also offer an interactive educational workshop at the end of the study for those who complete the follow-up.

#### Randomization

Participants will be randomized 1:1 to the intervention or control groups using the “Sealed Envelope” program (www.sealedenvelope.com), which provides high-quality and easy-to-use online software applications for randomizing patients into clinical trials using block randomization (blocks of 4, 6, and 8). Only the local study lead and nurse managers have access to randomization assignment. Other study investigators and data analysts will be blinded to treatment assignment. Data collectors will be blinded whenever feasible, although blinding cannot be guaranteed due to the nature of the intervention.

#### Study arms

All participants, regardless of group assignment, will be provided with medications prescribed by the local physician at their clinical discretion for the 6-month follow-up period. Individuals randomized to the intervention arm will receive the full case management intervention package. Individuals in the control group will receive the standard of care plus asthma education delivered by a nurse educator.

### Study outcomes

**Fig. 3:**
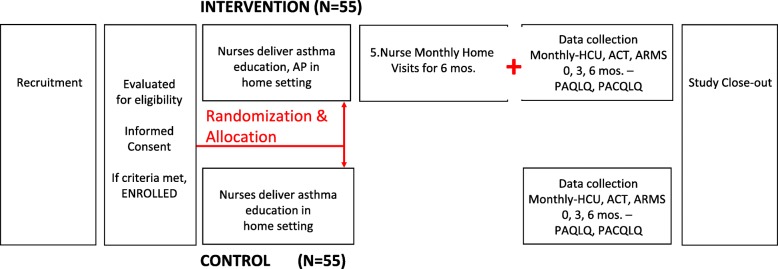
Participant timeline for the individually randomized trial

Trained independent data collectors will administer questionnaires either in person (baseline, 3 months, 6 months) or by phone (months 1, 2, 4, and 5). Details on the frequency and timeline for the following outcomes can be found in Table 2 and a participant timeline can be found in Fig. 3.

**Table 2:** Primary and secondary outcomes and frequency of assessment

Domain	Measurement method	Frequency
Asthma control	ACT score ED visits Hospitalizations	Monthly
Preventive healthcare utilization	Number of scheduled clinic visits	Monthly
Disease-specific quality of life	Pediatric Asthma Quality of Life Questionnaire	Baseline, 3 months, 6 months
Caregiver quality of life	Pediatric Caregiver Quality of Life Questionnaire	Baseline, 3 months, 6 months
Caregiver mental health	Patient Health Questionnaire (PHQ) - 9	Baseline, 3 months, 6 months
Medication uptake and adherence	ARMS-7 Inhaler counter Self-reported medication use	Monthly
Intervention engagement	Patient logs Text message transcripts	Throughout
Acceptability	In-depth interviews Focus group discussions Observations	Throughout
Feasibility	In-depth interviews Focus group discussions Observations Time logs	Throughout
Fidelity	Observations Audio recordings Checklists Visit logs	Throughout

*ACT* Asthma Control Test, *ED* Emergency Department

#### Effectiveness and adoption

##### Asthma control

We will measure asthma control at baseline and monthly using the Childhood Asthma Control Test (cACT) Score (ages 5–11 years) or Asthma Control Test (ACT) Score (ages 12–17 years) [13]. Both questionnaires are scored by summing the scores for all items. Lower scores indicate poorer asthma control; higher scores indicate better asthma control. Scores ≤ 19 for both questionnaires suggest impaired asthma control. The primary outcome will be ACT score at 6 months of follow-up.

##### Healthcare utilization

We will measure healthcare utilization (HCU) at baseline and monthly via self-report and medical records. Specific HCU outcomes will include proportion of children with an asthma-related emergency department (ED) or urgent care visits in the previous month, proportion with at least one asthma-related ED or urgent care visit during the 6-month follow-up period, number with all-cause hospitalizations, number with at least one all-cause hospitalization at 6 months, proportion with all-cause ED or urgent care visits during follow-up, and proportion with at least one all-cause ED or urgent care visit during the 6-month follow-up period.

##### Disease-specific quality of life

We will measure asthma quality of life in children and adolescents with asthma using the Pediatric Asthma Quality of Life Questionnaire (PAQLQ)-Mini [14]. The PAQLQ-mini has 13 items, each scored on a 7-point Likert scale with “1” indicating severe impairment and “7” indicating no impairment. The score (range: 1–7) is calculated by taking the average of scores for each item; higher scores indicate better quality of life.

##### Caregiver quality of life

We will measure the quality of life of caregivers using the Pediatric Asthma Caregiver’s Quality of Life Questionnaire (PACQLQ) [15]. The PACQLQ has 13 items, each scored on a 7-point Likert scale, with “1” indicating severe impairment and “7” indicating no impairment. The score (range: 1–7) is calculated by taking the average of scores for each item; higher scores indicate better quality of life.

##### Caregiver mental health

We will measure depressive symptoms among caregivers using the Patient Health Questionnaire-9 (PHQ-9). The PHQ-9 has nine items, each scored on a Likert scale of 0–3. Higher scores indicate more evidence of depressive disorder of greater severity of disorder; scores are calculated by summing the scores for all items. We will analyze differences in PHQ-9 score at baseline as compared to 6 months, as well as between treatment groups at 6 months.

##### Medication adherence

We will measure adherence to prescribed asthma medications at baseline and during follow-up visits. First, we will use the Adherence to Refills and Medications Scale-7 [16]. ARMS-7 be will be administered at baseline and monthly during the follow-up period. The score is calculated by summing the scores for all items; lower scores indicate better adherence. Second, we will measure adherence using inhaler counters placed on all daily inhaled medications (e.g. inhaled corticosteroids, long-acting beta agonist, combination therapies). We will evaluate differences in the number of puffs used divided by the number of puffs indicated to have been used by the prescribing physician over the monthly follow-up period.

#### Process evaluation

##### Fidelity to the intervention

We will conduct audio recordings and in-person observations of clinic staff, study staff, and study participants to evaluate fidelity (i.e. how well the intervention was delivered as intended according to the protocol). With participant consent, we will audio record all home visit interactions between nurse managers and families. Using pre-established checklists based on study protocols and standard operating procedures, we will evaluate the extent to which these procedures were followed by nurse managers in a random selection of 20% of audio files.

##### Acceptability and feasibility

We will conduct in-depth interviews and focus group discussions with approximately 30 child–caregiver dyads. These in-depth interviews and focus group discussions will inform understanding of the acceptability and feasibility of the intervention strategy from the perspectives of relevant stakeholders. We will also carry out in-depth interviews with nurse managers and local clinicians. Interviews will be semi-structured; we will have an interview guide but will also probe on relevant themes that emerge during the interview. We will also maintain detailed study records and carry out structured surveys to record information such as the frequency of contact by the participating family with the designated nurse manager, the amount of time spent by the nurse manager carrying out their duties as part of this intervention, and perceived satisfaction on the part of the participating families and nurse managers over time.

### Analytical plan

The primary effectiveness outcome is the mean difference in ACT scores at 6 months of follow-up between intervention and control groups. We will analyze these differences using t-tests; in subsequent analyses, we will use multivariable linear regression methods adjusting for baseline ACT score, and may also adjust for additional factors. The primary analysis will be carried out using an intention-to-treat approach. We will also carry out exploratory subset analyses of the difference in ACT score among individuals with high adherence to preventive therapies. In addition, we will use multivariable linear and logistic regression methods as appropriate to analyze differences in secondary outcomes.

#### Sample size

The primary objective of this pilot trial is to assess the feasibility, acceptability, and preliminary effectiveness of the intervention package in the Peruvian context. With a sample size of 100 participants, assuming a standard deviation of 3.6 and a type II error of 5%, we will be able to detect a difference of 2 in ACT score with 80% power. These calculations were based on preliminary data from a prior epidemiological study of children with asthma in this setting, in which the standard deviation of the ACT score was 3.6 [17]. In order to account for approximately 9% attrition, we arrived at a final sample size of 110 individuals.

### Data management and quality assurance

Questionnaire-based data will be collected using the Research Electronic Data Capture software (REDCap, Vanderbilt University Medical Center, Nashville, TN, USA). Data will be reviewed for accuracy and completeness on a weekly basis by two parties (data management team, study Principal Investigator [PI]).

To protect confidentiality, all participants will be assigned a unique identification code, which will allow data to be de-identified and stored without identifying information. Transcriptions and research notes will contain no identifying information. Pseudonyms for participants and locations will be used in all transcripts and research notes. Only members of the study team, including the PI and staff researchers, will have access to this data. Due to the low risks associated with this behavioral intervention, there are no pre-specified interim analyses or stopping rules.

### Adverse events

Due to the low risks associated with this behavioral intervention, we anticipate minimal occurrence of adverse events directly associated with the intervention. Nevertheless, adverse events occurring during follow-up (e.g. medication-related side effects) will be reported to the local study lead within 12 h of knowledge of their occurrence and to the study PI within 24 h. Serious adverse events (e.g. hospitalization, accident resulting in disability) will be reported to the local study lead within 6 h of learning of their occurrence and to the study PI within 12 h. Serious adverse events will be reported to the IRB committees within 1 week of the investigators becoming aware of the event.

### Dissemination

The results of this study will be published in peer-reviewed journals and submitted for presentation at international meetings. We will also disseminate results through reports and presentations to stakeholders within the Peruvian health system, as well as through workshops, online, and through social media to local communities.

## Discussion

This article presents the rationale, methods, and procedures for the AIRE research program, whose long-term goal is to promote adoption and access to self-management practices for children with asthma and their families in Lima, Peru and other LMIC settings.

The present study consists of an individually randomized implementation-effectiveness hybrid trial in which we will enroll 110 child–caregiver dyads affected by asthma in communities of Lima Norte. We will evaluate the effect of multi-component intervention strategy delivered with a case management approach on asthma control, disease-specific quality of life, medication adherence, and HCU, while also carefully evaluating process indicators to understand the factors that influence successful or unsuccessful implementation. The intervention is being carried out in the context of the Peruvian healthcare system with health professionals (nurses, physicians) with significant experience working in these local community health centers. While supported self-management interventions are a central piece of the puzzle, it is critical to concurrently identify potential solutions for addressing health system and policy level barriers, particularly those concerning the availability of affordable, quality-assured asthma medications.

This study builds upon several years of formative research efforts during which we have gathered and synthesized the perspectives of community members and local health practitioners regarding barriers and facilitators in this context. Team leadership includes nurses and physicians from within the communities of Lima Norte in which this study is taking place who have first-hand knowledge and authority with respect to the inner workings of the health system.

This study will contribute to the body of knowledge surrounding methods for developing and evaluating intervention strategies for asthma using systematic, theory-based approaches grounded in local context. Such strategies have the potential to inform the development and adaptation of feasible, appropriate, and scalable solutions for asthma management to bridge the gaps in care experienced disproportionately in LMIC settings.

### Trial status

The trial is ongoing. Enrollment began in June 2019 and is expected to be completed in March 2020. This study protocol reflects the approved version 1 from 10 January 2019.

## Data Availability

The datasets used and/or analyzed during the current study are available from the corresponding author on reasonable request.

## References

[1] Global Asthma Network (2018). The global asthma report 2018.

[2] Braman SS (2006). The global burden of asthma. Chest.

[3] Lai C, Beasley R, Crane J, Foliaki S, Shah J, Weiland S (2009). Global variation in the prevalence and severity of asthma symptoms: phase three of the International Study of Asthma and Allergies in Childhood (ISAAC). Thorax.

[4] Robinson CL, Baumann LM, Romero K, Combe JM, Gomez A, Gilman RH (2011). Effect of urbanisation on asthma, allergy and airways inflammation in a developing country setting. Thorax.

[5] National Asthma Education and Prevention Program (2007). Expert Panel Report 3 (EPR-3): guidelines for the diagnosis and management of asthma summary report 2007. J Allergy Clin Immunol.

[6] Global Initiative for Asthma. Global strategy for asthma management and prevention, 2019. www.ginasthma.org. Accessed 20 Feb 2020.

[7] Peru Ministry of Health (2006). Guías de práctica clínica para la atención de las patologías más frecuentes y cuidados esenciales del niño y la niña: Enfermedades respiratorias.

[8] Pinnock H, Parke HL, Panagioti M, Daines L, Pearce G, Epiphaniou E, Bower P, Sheikh A, Griffiths CJ, Taylor SJC, PRISMA and RECURSIVE groups (2017). Systematic meta-review of supported self-management practices for asthma: a healthcare perspective. BMC Med.

[9] Ahmed S, Steed L, Harris K, Taylor SJC, Pinnock H (2018). Interventions to enhance the adoption of asthma self-management behaviour in South Asian and African American populations: a systematic review. NPJ Prim Care Respir Med.

[10] Peru Ministry of Health. Recursos Humanos en Salud de Perú: Segundo Informe al País 2011. http://www.minsa.gob.pe/dggdrh/carrera_sanitaria/documentos/MANUALES%20E%20INFORMES/Recursos%20Humanos%20de%20Salud%20en%20Perú%202do%20Informe%20al%20Pa%C3%ADs.%20Marzo%202011.pdf. Accessed 20 Feb 2020.

[11] Michie S, van Stralen MM, West R (2011). The behaviour change wheel: A new method for characterising and designing behaviour change interventions. Implement Sci.

[12] SaludMadrid: Gerencia Asistencial de Atención Primaria. Guía de cuidados: Terapia Inhalada*.* 2016. http://www.madrid.org/cs/Satellite?blobcol=urldata&blobheader=application%2Fpdf&blobheadername1=Content-disposition&blobheadername2=cadena&blobheadervalue1=filename%3DGu%C3%ADa+de+cuidados+Terapia_Inhalada_ok_Junio_2016.pdf&blobheadervalue2=language%3Des%26site%3DPortalSalud&blobkey=id&blobtable=MungoBlobs&blobwhere=1352932248750&ssbinary=true. Accessed 20 Feb 2020.

[13] Schatz M, Kosinski M, Yarlas AS, Hanlon J, Watson ME, Jhingran P (2009). The minimally important difference of the Asthma Control Test. J Allergy Clin Immunol.

[14] Juniper EF, Guyatt GH, Feeny DH, Ferrie PJ, Griffith LE, Townsend M (1996). Measuring quality of life in children with asthma. Qual Life Res.

[15] Juniper EF, Guyatt GH, Feeny DH, Ferrie PJ, Griffith LE, Townsend M (1996). Measuring quality of life in the parents of children with asthma. Qual Life Res.

[16] Kripalani S, Risser J, Gatti ME, Jacobson TA (2009). Development and evaluation of the Adherence to Refills and Medications Scale (ARMS) among low-literacy patients with chronic disease. Value Health.

[17] Pollard SL, Lima JJ, Romero K, Tarazona-Meza C, Mougey E, Tomaino K, Malpartida-Guzmán G, Hansel NN, Checkley W (2017). Associations between serum 25(OH) D concentrations and prevalent asthma among children living in communities with differing levels of urbanization: a cross-sectional study. Asthma Res Pract.

